# Utilizing deep learning algorithms and artificial neural networks to forecast the viscosity, thermal conductivity, and electrical conductivity of Fe_3_O_4_/TiO_2_ magnetic hybrid nanofluid

**DOI:** 10.1038/s41598-026-45886-3

**Published:** 2026-04-06

**Authors:** Shadha K. Jebur, Hiba M. Abdullah, Areej. D. Abbas, Narinderjit Singh Sawaran Singh, Dheyaa J. Jasim, Soheil Salahshour, A. Rahimi

**Affiliations:** 1 College of Chemical Engineering, University of Technology- Iraq, Baghdad, 10066 Iraq; 2 Faculty of Data Science and Information Technology, INTI International University, Persiaran Perdana BBN, Putra Nilai, Nilai, 71800 Malaysia; 3 College of Engineering, University of Al Maarif, Al Anbar, 31001 Iraq; 4 Faculty of Engineering and Natural Sciences, Istanbul Okan University, Istanbul, Turkey; 5 Faculty of Engineering and Natural Sciences, Bahcesehir University, Istanbul, Turkey; 6 Research Center of Applied Mathematics, Khazar University, Baku, Azerbaijan; 7 Faculty of Science and Letters, Piri Reis University, Tuzla, Istanbul, Turkey; 8 Fast Computing Center, Shabihsazan Ati Pars, Tehran, Iran

**Keywords:** Machine Learning; Energy access, Hybrid Nanofluid Fe₃O₄/TiO₂, Multi-objective Optimization, Thermophysical Properties, Energy science and technology, Engineering, Mathematics and computing, Nanoscience and technology

## Abstract

Machine learning provides a powerful approach for predicting the complex thermophysical properties of nanofluids. This study employs a suite of machine learning algorithms to forecast the viscosity, thermal conductivity, and electrical conductivity of a Fe₃O₄/TiO₂ magnetic nanofluid, using experimental data over the temperature range of 10–50 °C and volume fractions of 0–0.3%. Among Gaussian Process Regression, Multiple Linear Regression, Support Vector Regression, Multilayer Perceptron, and Multiple Polynomial Regression (MPR), the MPR model demonstrated superior performance, achieving a correlation coefficient above 0.99 and the lowest error metrics (e.g., Root Mean Square Error of 0.0216 for viscosity). Subsequent multi-objective optimization using the Multi-objective Grey Wolf Optimizer (MOGWO) generated a Pareto front of optimal solutions. The most balanced solution, identified using entropy-based weighting, corresponded to a configuration of 60 wolves and 300 iterations. This integrated framework accurately predicts the thermophysical properties and identifies optimal trade-offs for engineering applications.

## Introduction

Enhancing heat transfer efficiency in energy systems and thermal exchangers is a critical challenge in thermal engineering, directly impacting energy consumption, operational costs, and environmental sustainability^1^. In industrial settings such as power plants and electronic cooling systems, improved heat transfer coefficients enable downsized equipment, extended device lifetimes, and reduced energy waste. To address these needs, innovative technologies such as nanofluids and machine learning (ML) algorithms have emerged as transformative solutions. Nanofluids—engineered by dispersing nanoparticles in base fluids—offer exceptional potential to enhance convective heat transfer due to their tunable thermophysical properties. The foundational study by Choi and Eastman^2^ showed that nanoparticles could substantially improve the thermal conductivity of base fluids. This has been further validated by systems such as copper-based nanofluids, which are effective for high-power cooling^3^. Nonetheless, broader application of nanofluids requires overcoming challenges related to colloidal stability and increased viscosity. Modern thermal system design benefits from intelligent modeling approaches. Machine learning algorithms, for instance, help comprehend complex fluid dynamics and identify optimal operational configurations. Ghorbani and Ranjbar^4^, for instance, demonstrated that applying genetic algorithms to optimize heat exchanger flow parameters can increase thermal efficiency by up to 15%. This illustrates the potential of merging nanofluid research with machine learning to create comprehensive solutions for heat transfer challenges. Such an integrated approach can enable the development of new sustainable energy systems and advanced thermal management technologies. Furthermore, predictive models, such as artificial neural networks, trained on experimental nanofluid data have successfully aided in the design of high-efficiency cooling systems^5^.

Nanofluids have revolutionized energy engineering and thermal systems as advanced heat transfer media. They are produced by dispersing nanoparticles—including metals (e.g., Cu, Ag), metal oxides (e.g., Al₂O₃, TiO₂, CuO), carbon nanotubes, and graphene—into base fluids like water, ethylene glycol, or oil. This dispersion significantly enhances thermal conductivity and convective heat transfer coefficients^6^. However, practical challenges such as nanoparticle stability, increased viscosity, and higher costs limit their widespread implementation^7^. To overcome these limitations, hybrid nanofluids incorporating two or more nanoparticle types (e.g., Al₂O₃-Cu, SiO₂-MWCNT) have been developed^8^. By combining the advantages of individual particles, hybrid systems achieve superior thermal conductivity, greater stability, and better viscosity control than single-particle nanofluids. For instance, combining metallic nanoparticles with carbon nanotubes can enhance both thermal and mechanical properties. Developing these hybrids requires a deep understanding of inter-nanoparticle interactions and their effect on thermal performance under various conditions. Although production costs are higher than those of conventional nanofluids, hybrid nanofluids offer a promising solution for demanding industrial applications, such as advanced heat exchangers and cooling systems, where maximum thermal efficiency is critical^9^.

An innovative approach to further enhance nanofluid performance involves applying external magnetic fields, which improve fluid management and heat transfer rates through Lorentz forcing and intensified turbulence. Among magnetic nanoparticles, Fe₃O₄ (magnetite) is a prime candidate due to its strong ferromagnetism, colloidal stability, and thermal efficiency. A key benefit is that the magnetic field promotes uniform nanoparticle distribution, preventing aggregation and enhancing thermal performance. Experimental studies demonstrate these effects, reporting significant enhancements in thermal conductivity in various Fe₃O₄-based nanofluids^10,11^. For thermal applications, TiO₂ nanoparticles are also highly attractive due to their complementary properties: good chemical stability, corrosion resistance, and photocatalytic activity. Therefore, combining Fe₃O₄ and TiO₂ yields a smart hybrid nanofluid that integrates magnetically controllable heat transfer with long-term stability and self-cleaning properties This synergy is promising for advanced heat exchangers. Recent studies confirm the benefits of this hybrid system. For instance, improved thermal performance has been reported in oil-based Fe₃O₄/TiO₂ nanofluids^12^, extended operational life in transformer oils^13^, and significant thermal conductivity gains in ethylene glycol^14^ and hydraulic oil^15^ based hybrids, with enhancements strongly dependent on nanoparticle concentration and temperature. Beyond thermal conductivity, the stability and rheology of these hybrids are critical. For example, Shahsavar et al.^15^ confirmed high colloidal stability via zeta potential measurements, while Sepehrnia et al.^16^ reported dramatic, temperature-sensitive viscosity increases in oil-based Fe₃O₄/TiO₂/GO hybrids, highlighting the importance of formulation optimization. The heat transfer performance of Fe₃O₄/TiO₂ nanofluids in practical configurations is particularly promising. Sundar et al.^17^ demonstrated that applying a magnetic field (up to 1000 Gauss) to a mini-heat sink could boost the Nusselt number enhancement from 38.16% to 88.93% compared to water. Adogbeji et al.^18,19^ further explored this system, showing enhanced thermal performance in tubes under magnetic fields, albeit with a dependence on the field waveform. Their work also revealed a key engineering trade-off: while a low concentration (0.0125% vol.) yielded a 26.33% increase in heat transfer in turbulent flow, higher concentrations increased pressure drop by up to 18.7%^19^. These studies collectively underscore the potential of Fe₃O₄/TiO₂ hybrid nanofluids as smart, magnetically tunable heat-transfer media. However, they also reveal persistent challenges: managing viscosity spikes, optimizing magnetic-field application, and balancing heat-transfer gain with a pumping-power penalty. Furthermore, high production costs and the need for specialized magnetic control equipment remain hurdles for widespread industrial adoption.

The engineering sciences field has undergone a paradigm shift with the advent of machine learning (ML) tools, which enable the analysis of complex datasets, predictive modeling, and parameter optimization across fields such as materials science and nanotechnology^20^. Research on hybrid nanofluids is particularly challenging, as conventional modeling struggles to capture the nonlinear behavior arising from particle interactions, distribution, and size^21^. Here, ML techniques such as artificial neural networks (ANNs) and support vector machines (SVMs) offer transformative solutions by extracting hidden patterns from experimental data to efficiently predict properties like thermal conductivity, viscosity, and stability. However, the application of advanced ML remains underutilized for Fe₃O₄/TiO₂ hybrid nanofluid systems. It is worth noting that for other hybrid nanofluid systems—such as Al₂O₃–Cu for thermal conductivity and specific heat prediction using artificial neural networks^22,23^, Al₂O₃-CuO, Al₂O₃-TiO₂, and for thermal applications like battery cooling and solar collectors, the integration of machine learning for prediction and optimization has seen significant recent activity^24–27^. While some studies have employed ML for specific tasks on Fe₃O₄/TiO₂—such as predicting density and viscosity^28^ or dynamic viscosity^16^—a comprehensive comparative analysis of multiple ML algorithms, integrated with multi-objective optimization for trade-off analysis, is still lacking for this system.

Therefore, this study introduces a novel, integrated framework for the Fe₃O₄/TiO₂ system. We utilize the experimental dataset from^19^ to benchmark five distinct ML models: Gaussian Process Regression, Multiple Linear Regression, Support Vector Regression, Multilayer Perceptron, and Multiple Polynomial Regression. Following the identification of the optimal predictive model (MPR), we integrate it with the Multi-objective Grey Wolf Optimizer (MOGWO) to identify Pareto-optimal solutions that balance the competing objectives of minimizing viscosity while maximizing thermal and electrical conductivity. Finally, we employ the TOPSIS decision-making method to select the best operating point based on application-specific priorities, considering different weighting scenarios. This work not only delivers a validated predictive and optimization tool for Fe₃O₄/TiO₂ nanofluids but also presents a generalizable methodology for analyzing and designing advanced heat transfer fluids.

## Methodology

The thermophysical properties of nanofluids—including viscosity, thermal conductivity, and electrical conductivity—are primarily influenced by temperature and nanoparticle volume fraction. As with conventional fluids, viscosity decreases with increasing temperature due to reduced intermolecular adhesion. Increasing the nanoparticle volume fraction introduces more solid particles into the fluid, thereby increasing resistance to flow and raising viscosity. Increased temperature reduces intermolecular adhesion, lowering resistance to flow. On the other hand, as the volume fraction of nanoparticles increases, the viscosity also increases because more solid particles in the fluid increase the resistance to fluid flow. Some studies, including that of Giwa et al.^29^, have observed this behavior, showing that viscosity increases dramatically with increasing nanoparticle volume fraction. Nanoparticle concentration and temperature affect the thermal conductivity of nanofluids. This characteristic is enhanced as the volume fraction of the nanoparticles and the temperature increase. Due to the increased surface contact between solid particles and the base fluid, as well as the enhanced heat transfer of the nanoparticles, the thermal conductivity of the fluid increases with the addition of nanoparticles. As temperatures rise, particles’ kinetic energy increases, thereby enhancing heat transfer. At the same time, Fe_3_O_4_/TiO_2_ hybrid magnetic nanofluids exhibited higher thermal conductivity compared to deionized water, as indicated by experimental data. This level of conductivity became more pronounced at lower nanoparticle volume fractions and higher temperatures^30^. Electrical conductivity also depends on the volume fraction of nanoparticles and temperature. As the volume fraction increases, the nanoparticles form a more percolative network within the fluid, creating additional pathways for electron transport and thereby increasing conductivity. With rising temperature, the increased kinetic energy of charge carriers (ions and electrons) enhances their mobility. Simultaneously, the reduction in the fluid’s internal resistance due to decreased viscosity further facilitates charge transfer, leading to a net increase in electrical conductivity^29^. This behavior has been confirmed in various studies, which indicate that hybrid magnetic nanofluids have the potential to improve electrical conductivity.

### Machine learning algorithm

In recent years, machine learning (ML) has emerged as a transformative approach for modeling the thermophysical properties of nanofluids, capable of capturing complex, non-linear relationships that challenge conventional correlations. By learning directly from experimental data, ML algorithms can provide accurate predictions for engineering design and optimization. This study implements and compares five established ML regression algorithms, selected for their proven efficacy and complementary approaches to predictive modeling. Hyperparameters for all models were optimized using a grid search combined with 5-fold cross-validation on the training data, selecting the configuration that minimized the root mean square error (RMSE). The final models were then evaluated using repeated random subsampling validation, reported as mean ± standard deviation.

**Support Vector Regression (SVR)** is a supervised learning algorithm adapted from Support Vector Machines for regression tasks. Its core principle is to find a function that deviates from the observed data by no more than a defined tolerance margin, while remaining as flat as possible^31,32^. This formulation, often coupled with kernel functions (such as the radial basis function) to handle nonlinearity, makes SVR notably robust to outliers—a valuable trait when working with experimental datasets that may contain noise. A primary consideration is its computational scaling with dataset size.

**Gaussian Process Regression (GPR)** is a probabilistic, non-parametric model that places a Gaussian process prior over functions. Instead of predicting a single value, GPR provides a full predictive distribution, yielding both an estimate and a quantitative measure of uncertainty (confidence intervals)^33^. This ability to explicitly model uncertainty is particularly advantageous for scientific and engineering applications, such as predicting nanofluid properties, where understanding the reliability of a prediction is as important as the prediction itself. The method’s flexibility can demand greater computational resources.

**A Multilayer Perceptron (MLP)** is a class of feedforward artificial neural networks consisting of multiple layers of interconnected neurons. Each neuron applies a non-linear activation function, allowing the network to learn and represent highly complex, hierarchical patterns in data^34^. This universal approximation capability makes MLPs powerful for modeling the intricate dependencies of nanofluid properties on temperature and concentration. Their main practical challenge lies in designing the network architecture and applying regularization techniques to prevent overfitting, especially with limited data.

**Multiple Linear Regression (MLR)** models the relationship between several independent variables and a dependent variable by fitting a linear equation. It is highly interpretable, as the coefficient of each input variable quantifies its influence on the output^35,36^. We include MLR as a fundamental baseline model. Its performance serves as a critical reference point; if more complex algorithms do not significantly outperform MLR, it suggests the underlying relationships may be predominantly linear within the studied domain.

**Multiple Polynomial Regression (MPR)** extends MLR by incorporating polynomial terms (e.g., squares, cubes, interaction terms) of the independent variables. This allows the model to capture curvilinear trends and interaction effects that are common in physical systems, such as the synergistic effects between temperature and nanoparticle concentration on viscosity or conductivity^35,36^. MPR offers a structured middle ground between rigid linear models and highly flexible black-box models. The key to its successful application is carefully selecting the polynomial degree to balance model expressiveness with the risk of overfitting The comparative analysis of these five algorithms—from simple and interpretable to complex and flexible—allows us to rigorously identify the most suitable predictive model for the Fe₃O₄/TiO₂ hybrid nanofluid system while providing insights into the nature of the underlying property relationships.

### Evolution criteria

A comprehensive evaluation of predictive model performance requires multiple complementary metrics, as each captures different aspects of error and fit. We employ a suite of seven standard metrics to provide a robust, multi-faceted assessment. The rationale for selecting each is as follows.

The correlation coefficient (R) and the coefficient of determination (R²) are fundamental for assessing the strength and direction of the linear relationship, and the proportion of variance explained by the model, respectively. While related, both are reported to give a complete picture of explanatory power^35,36^. To quantify prediction error, we use three core metrics with distinct sensitivities. The Mean Squared Error (MSE) and its square root, the Root Mean Squared Error (RMSE), are sensitive to large errors due to squaring, making them crucial for identifying significant deviations. In contrast, the Mean Absolute Error (MAE) provides a more direct average of absolute errors, treating all deviations equally and offering a robust view when outliers are not the primary concern. For scale-independent comparison—essential when evaluating properties with different units, such as viscosity and conductivity—we use percentage-based errors. The Mean Absolute Percentage Error (MAPE) expresses accuracy as a percentage of the actual values, while the Mean Relative Absolute Error (MRAE) normalizes error relative to a simple benchmark, highlighting the model’s improvement over a basic baseline. Collectively, these seven metrics evaluate model performance from multiple angles: explanatory power (R, R²), error magnitudes at different outlier sensitivities (MSE, RMSE, MAE), and scale-normalized accuracy (MAPE, MRAE). Their mathematical definitions are provided in Eq. (1) through (7).

1 $$\:\mathrm{R}=\frac{{\sum\:}_{i=1}^{n}\left({y}_{i,Experiment}-{\stackrel{-}{y}}_{Experiment}\right)\left({y}_{i,predict}-{\stackrel{-}{y}}_{predict}\right)}{\sqrt{{\sum\:}_{i=1}^{n}{\left({y}_{i,Experiment}-{\stackrel{-}{y}}_{Experiment}\right)}^{2}{\sum\:}_{i=1}^{n}{\left({y}_{i,predict}-{\stackrel{-}{y}}_{predict}\right)}^{2}}}$$

2 $$\:RMSE=\sqrt{\frac{\sum\:_{i=1}^{n}{({y}_{i,predict}-{y}_{i,Experiment})}^{2}}{n}}$$

3 $$\:MAE=\frac{\sum\:_{i=1}^{n}\left|{y}_{i,predict}-{y}_{i,Experiment}\right|}{n}$$

4 $$\:MSE=\frac{\sum\:_{i=1}^{n}{({y}_{i,predict}-{y}_{i,Experiment})}^{2}}{n}$$

5 $$\:{R}^{2}=1-\:\frac{\sum\:_{i=1}^{n}{({y}_{i,predict}-{y}_{i,Experiment})}^{2}}{\sum\:_{i=1}^{n}{({y}_{i,Experiment}-{\stackrel{-}{y}}_{Experiment})}^{2}}$$

6 $$\:MAPE=\frac{\sum\:_{i=1}^{n}\left|\frac{{y}_{i,predict}-{y}_{i,Experiment}}{{y}_{i,Experiment}}\right|}{n}*100\:\:\:$$

7 $$\:MRAE=\frac{1}{n}*\:\:\sum\:_{i=1}^{n}\left|\frac{{y}_{i,predict}-{y}_{i,Experiment}}{{y}_{i,Experiment}}\right|$$

The Taylor diagram is a sophisticated graphical instrument utilized by statisticians and geoscientists to assess the precision of various predictive models. This method presents data in a way that simultaneously displays three critical statistical parameters: standard deviation, correlation, and root-mean-square deviation (RMSD). This facilitates an immediate evaluation and comprehension of how closely the model output aligns with the actual data. It enables plotting multiple models or datasets on a single polar map, allowing concurrent comparison of several metrics. This approach enables a direct comparison of results, highlighting potential areas for improvement. The Taylor diagram provides a graphical method for visualizing and quantifying the degree of correspondence between expected and observed data. Every point on that diagram represents the performance of a different dataset (or model) against the real data. The angular position of the point indicates the degree of overlap between the crafted model’s predictions and the data, and the radial distance indicates the spread. A key metric that comprises the overall error is the Root Mean Square Deviation (RMSD), along with the distance between the model point and the reference point. The Taylor diagram combines three statistics to objectively compare the individual merits of multiple models, thereby simplifying the evaluation of complex model performance. The Taylor diagram is especially useful for visually comparing the accuracy of previous predictions to observed data. Different datasets (or models) are represented by points on the graph, which plots their effectiveness relative to the original data. The radial distance indicates dispersion, whereas the angular position of the point represents the accuracy of the model’s predictions compared to the actual data.

Root-mean-squared deviation (RMSD) is a crucial metric because it is population-measurable and quantifies overall imprecision by measuring the distance between a point in the model and its corresponding point in the reference. The Taylor diagram combines these three signals into a single measure, ultimately allowing the assessment of complex model performance and the relative advantage of one model over another. One reason for the swift rise in the popularity of the Taylor diagram might be its simplicity. This image combines standard deviation, correlation, and RMSD into a single visualization, allowing model performance to be represented without complex numerical analysis. It is designed to be intuitive for researchers, legislators, and stakeholders with little or no statistical training. It is an image that offers extensive configurability, presenting research opportunities. It may elicit more comments or adjustments to dimensions, with a focus on key performance metrics. Equations (8) and (9) harvest the RMSD indices and standard deviation.

8 $$\:{{\sigma\:}_{ANN,Exp}}^{2}=\frac{1}{N}\sum\:_{n=1}^{N}{\left({{y}_{pred,Exp}}^{\left(i\right)}-\stackrel{-}{{{y}_{pred,Exp}}^{\left(i\right)}}\right)}^{2}$$

9 $$\:{RMSD}^{2}={{\sigma\:}_{Exp}}^{2}+{{\sigma\:}_{pred}}^{2}-2\:\:{\sigma\:}_{Exp}{\sigma\:}_{pred}R$$

### Multi-objective Grey Wolf Optimizer (MOGWO)

The Multi-objective Grey Wolf Optimizer (MOGWO), a nature-inspired metaheuristic algorithm, was introduced for solving multi-objective optimization problems. This particular algorithm was proposed by Mirjalili et al.^37^, inspired by the social behavior of grey wolves observed in nature and their hierarchical structure. Based on this concept, wolves represent implicit candidate solutions that are simulated through hunting actions, such as circling, coursing, and attacking, thereby efficiently exploring the search space. Due to its multi-objective nature, MOGWO handles solutions using techniques such as Pareto solution sets and non-dominated sorting, unlike the single-objective variant of the Grey Wolf Optimizer (GWO)^38^. This feature enables the algorithm to generate a set of Pareto-optimal candidates, from which a decision-maker can select the most suitable solution that aligns with their conflicting objectives. A significant application of MOGWO is found in fields such as industrial engineering, energy system design, water resource management, and neural network parameter optimization, where multi-objective optimization is crucial^39^. Furthermore, MOGWO is adept at addressing complex problems characterized by high dimensionality, thanks to its straightforward structure and ability to generate a variety of solutions. Nonetheless, significant challenges stem from the algorithm’s high computational complexity, particularly when the problem size and the number of objectives are substantial, leading to prolonged execution times. Suggested approaches to mitigate this challenge involve integrating dimensionality reduction methods and enhancing selection and search strategies within the framework^40^. Narrowly, as one of the important tools in the researchers’ toolbox for optimization, MOGWO plays a crucial role in the scientific and applied developments of Multi-objective optimization problems^37^. The change values for the MOGWO algorithm are shown in Table 1.

**Table 1:** MOGWO parameter combinations.

Parameter	Values
Number of wolves	50, 60, 70
Number of iterations	100, 200, 300, 400, 500
Archive size	100
Grid resolution	10 divisions per objective
Random seed	42 (fixed)

### Evaluation methods of the multi-objective optimization algorithm

This study selected three indicators—the quantity of Pareto solutions, the average ideal distance, and the duration—from numerous options to determine a suitable multi-objective optimization method.

#### Mean Ideal Distance (MID)

The Mean Ideal Distance (MID) index measures the average distance between the ideal solution and the Pareto points generated by the algorithms. The optimal solution identified in the study incorporates all techniques and represents the highest achievable value for each Objective Function^41^. The significance of this metric is evident: a lower value indicates greater algorithmic efficiency. In conclusion, it suggests that algorithms perform better when the mean distance between the Pareto points and the optimal solution is minimized, indicating that the solutions produced are closer to the most effective options. Equation (10) outlines the formula used to compute the MID index.

10 $$\:MID=\frac{\sum\:_{i=1}^{n}\sqrt{{{f}_{1i}}^{2}+{{f}_{2i}}^{2}+{{f}_{3i}}^{2}+{{f}_{4i}}^{2}}}{n}$$

As mentioned earlier, ‘n’ represents the number of non-dominated solutions in a set. The initial, second, third, and fourth objective values of the ‘*i*-th’ non-dominated solution in the set are denoted by *f*_*1i*_, *f*_*2i*_, *f*_*3i*_, and *f*_*4i*_, respectively. This formula, focusing on the first and second objectives, establishes a relevant metric by considering the number of non-dominated solutions and their corresponding objective values.

#### 2.4.2 Algorithm execution time index (Time)

A key metric for comparing algorithms is this index, which assesses their execution time. A lower value for this index indicates improved algorithmic efficiency, provided that all other factors remain unchanged. Reducing execution time not only enhances the algorithm’s efficiency but also accelerates the generation of results. This is typically advantageous, but it becomes particularly beneficial when time is of the essence in decision-making.

#### Spacing (SP)

Schott was the originator of the spacing metric^42^. The primary objective of this metric was to assess the variance of the identified Pareto-optimal solutions. The subsequent mathematical expression of SP^43^ is as follows:

11 $$\:SP=\sqrt{\frac{1}{\left|n\right|}\sum\:_{i=1}^{n}{\left({d}_{i}-\stackrel{-}{d}\right)}^{2}}$$

Let *n* be the total count of Pareto-optimal solutions identified, and d denote the mean of all $\:{d}_{i}$ values. The subsequent expression represents the mathematical expression for $\:{d}_{i}$.

12 $$\:{d}_{i}=\begin{array}{c}\mathrm{m}\mathrm{i}\mathrm{n}(\left|{{f}^{i}}_{1}\left(\overrightarrow{x}\right)-{{f}^{j}}_{1}\left(\overrightarrow{x}\right)\right|+\left|{{f}^{i}}_{2}\left(\overrightarrow{x}\right)-{{f}^{j}}_{2}\left(\overrightarrow{x}\right)\right|+\left|{{f}^{i}}_{3}\left(\overrightarrow{x}\right)-{{f}^{j}}_{3}\left(\overrightarrow{x}\right)\right|+\left|{{f}^{i}}_{4}\left(\overrightarrow{x}\right)-{{f}^{j}}_{4}\left(\overrightarrow{x}\right)\right|\\\:j\end{array}\:i,j=\mathrm{1,2},\dots\:,n\:$$

A low value for this metric indicates a more uniform distribution of options along the Pareto-optimal frontier.

#### Maximum Spread (MS)

Zitzler^44^ introduced this metric, known as the variety. Equation (13) represents the formula for the MS metric, utilized to compute the diagonal length of a hyperbox formed by the extreme function values identified on the Pareto curve.

13 $$\:MS=\sqrt{\sum\:_{i=1}^{o}\mathrm{m}\mathrm{a}\mathrm{x}\left(\right(d\left({a}_{i},{b}_{i}\right))}$$

where $\:{a}_{i}$ represents the maximum value in the ith goal, $\:{b}_{i}$ denotes the minimum value in the ith objective, and *o* signifies the total number of objectives. $\:d\left({a}_{i},{b}_{i}\right)$ calculates the Euclidean distance.

This equation illustrates how this metric computes the maximum diagonal distance and uses the identified Pareto-optimal front to construct a hyperbox (hypercube).

### Assessment methods for ranking multi-objective optimization algorithms

#### Shannon’s entropy method

Shannon’s entropy method^45^ is an objective weighting technique based on information theory. It measures the dispersion or uncertainty in each criterion’s values across alternatives. Criteria with lower entropy (more structured data) receive higher weights, as they provide more useful information for decision-making^46^. This approach is used in fields such as social science, engineering, economics, and environmental management to rank criteria by determining their relative importance in the decision-making process. Shannon’s entropy is used as a measure of complexity to quantify the uncertainty in a given dataset. The Criterion Entropy: The entropy of a criterion is the degree of uncertainty regarding the values of a criterion over all the alternatives, and is directly involved in the decision-making process. Lower entropy numbers indicate that the data is more compact or structured, while higher entropy values suggest that the data is more evenly distributed or random. Shannon’s entropy method is used in index weighing methods to determine the weight of each criterion based on the information it provides. The fundamental premise of the technique is to assign greater significance to factors that substantially influence the decision-making process, indicating they possess lower entropy or more condensed information. In contrast, criteria exhibiting greater entropy and a more uniform distribution are afforded less significance, as they provide less information.

#### TOPSIS method

The Technique for Order of Preference by Similarity to Ideal Solution (TOPSIS) is a widely used multi-criteria decision-making method that facilitates the selection and ranking of alternatives based on various criteria. This straightforward algorithm, introduced by Hwang and Yoon^47^, identifies the option closest to the positive ideal solution while farthest from the negative ideal solution, thereby establishing a reference point for evaluation. In this approach, the criteria and options are first numerically normalized to a common scale for comparison. Then, weights are assigned to the requirements, indicating the importance of each criterion in the decision-making process. After calculating the distance of each option from the positive and negative ideal solutions, a final score is determined for each option, which can be used to rank the options^48^. One of the important features of TOPSIS is its simplicity and interpretability, which allows users to examine the decision-making process transparently. In addition, TOPSIS has been applied in various fields, including project management, supplier selection, performance evaluation, and financial decision-making, due to its flexibility in handling different criteria and its ability to integrate quantitative and qualitative data^49^. However, this algorithm is highly dependent on the exact weighting of the criteria, which can vary depending on decision-makers’ thinking; this is one of its major drawbacks. A variety of methods, such as entropy-based or AHP-based objective weighting, have been proposed to address this problem^46^. As one of the most widely used tools in multi-criteria decision-making research, TOPSIS has made significant contributions to scientific and practical advances in evaluation and ranking problems^47^.

## Experiment and analysis

### Data preparation

This study incorporates the method and findings of Adogbeji et al.^19^. The Fe_3_O_4_/TiO_2_ hybrid NFs flow was thoroughly investigated in their thermophysical property studies. Important metrics, such as TC, DV, and EC, are tested within a temperature range of 10–50 °C and a volume fraction range of 0–0.3.3%. The chosen temperature range of 10–50 °C covers common operating conditions for many industrial cooling systems and electronic thermal management applications. The volume fraction range of 0–0.3% is strategically selected as it represents a critical window for hybrid nanofluids: it is sufficiently high to elicit measurable enhancements in thermophysical properties, yet low enough to mitigate the severe increases in viscosity and aggregation risks that typically occur at higher nanoparticle loadings, thereby maintaining colloidal stability and practical pumpability^7,19^. These ranges provide a relevant and experimentally viable domain for modeling the performance of the Fe₃O₄/TiO₂ hybrid nanofluid. To be more specific, 72 points were taken from Adogbeji et al.‘s experimental dataset^19^. Because it is precise and enhances the validity of the optimization, the modeling technique plays a crucial role in this study^50,51 [52,53]^. The first step in developing AI models is to identify the elements that influence each response. Several factors regarding the NPs and the base fluid must be considered, as the NF’s TPPs may be affected in both significant and minor ways. Previous studies have highlighted several features as critical for distinguishing various NF TPPs^46,47^. Their findings prompted further exploratory studies to identify the factors impacting the TPPs in the present data. The models are simplified, and the analysis is made more efficient and of higher quality using this technique. Two variables that could affect viscosity are temperature and the fraction of NP volume. Using the correct surfactant in conjunction with solid NPs added to a liquid allows one to manipulate the rheological properties. The use of plant-based surfactants to enhance dewatering behavior and reduce moisture content in iron ore fines has shown promising results. In addition, research has shown that these surfactants improve the ore grade by lowering the gangue concentration of the fines^53,54,55^. The complete dataset used in this study has been provided as Supplementary Material (Dataset_S1.csv). The file contains 72 rows and 5 columns: temperature T (°C), volume fraction φ (%, used as percentage), dynamic viscosity DV (mPa·s), thermal conductivity TC (W/m·K), and electrical conductivity EC (mS/cm). No scaling or normalization was applied to any variable. All models were trained on the raw data in their original units. For initial model development and hyperparameter tuning, the dataset was randomly split into training (80%, 58 points) and test (20%, 14 points) sets using a fixed random seed (42) to ensure reproducibility. To ensure robust model evaluation given the limited dataset size, we further employed repeated random subsampling validation with 10 repetitions. In each repetition, a new random 80/20 split was generated using different random seeds (42 to 51). All five ML models were retrained on the corresponding training set, and all seven performance metrics were calculated on the corresponding test set. For each model and each metric, the mean and standard deviation were computed across the 10 repetitions. The results are presented in Table 3(a-c) as mean ± standard deviation, providing a robust statistical basis for model comparison.

### Results

**Fig. 1:**
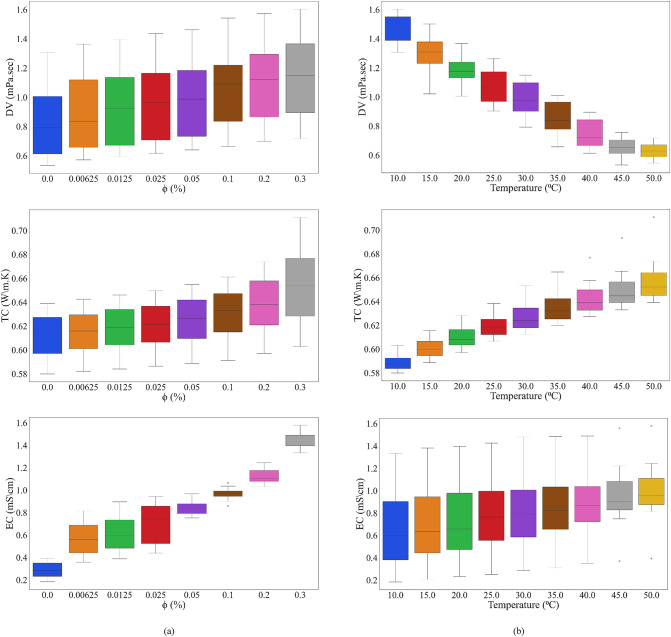
Effects of changing input factors on NF’s thermophysical characteristics.

This study aimed to explore the role of two input parameters: temperature (°C) and the volume fraction of nanoparticles (φ (%)), on three outputs, dynamic viscosity (DV (mPa. s)), thermal conductivity (TC (W/m.K)) and electrical conductivity (EC (mS/cm)). As shown in Fig. 1, the temperature and nanoparticle volume fraction significantly affect the nature of these thermophysical properties. With increasing temperature, dynamic viscosity decreases due to reduced intermolecular adhesion and reduced flow resistance. On the other hand, increasing the volume fraction of nanoparticles increases viscosity because more solid particles in the fluid increase flow resistance. For thermal conductivity, this property rises with increasing temperature and nanoparticle volume fraction. This is due to a larger contact area and better heat conduction from the hot particles, resulting from higher kinetic energy at higher temperatures. This also indicates that electrical conductivity increases as the volume fraction of nanoparticles and the temperature increase. This is attributed to the formation of conductive networks by nanoparticles, which facilitate electron transport, and to the reduction in internal resistance with increasing temperature. Therefore, in Figs. 1(a) and 1(b), the thermophysical properties of nanofluids are influenced by changes in both temperature and nanoparticle volume fraction, and these effects differ across the properties (viscosity, thermal conductivity, and electrical conductivity). Overall, this study demonstrates that appropriate selection of temperature and nanoparticle volume fraction can enhance nanofluid performance across various applications.

The effort’s declared objective is to use a machine learning system to forecast the outcomes of laboratory trials. Machine learning techniques, including GPR, MLR, SVR, MLP, and MPR, were employed to achieve this goal. The algorithm receives the 5*72 dataset, with the input (T, φ) in the first two columns and the objective function or output (DV, TC, EC) in the final three columns. All machine learning models were implemented in MATLAB R2022a using appropriate toolboxes. No scaling or normalization was applied to input or output variables, preserving the physical interpretability of the models. The configuration details for all five algorithms are summarized in Table 2.

**Table 2:** Configuration details of machine learning models implemented in MATLAB.

Model	MATLAB Function/Toolbox	Key Parameters	Architecture Details	Training Optimization Settings	Stopping Criteria
MLR	fitlm (Statistics Toolbox)	Formula: y ~ T + φ, Intercept: Included	N/A	Ordinary Least Squares (analytical solution)	N/A (closed-form solution)
MPR	fitlm with custom polynomial matrix (Statistics Toolbox)	Degree: 5, All interaction terms included	Feature space: All T and φ combinations up to degree 5 (21 features)	Ordinary Least Squares	N/A (closed-form solution)
SVR	fitrsvm (Statistics Toolbox)	Kernel: RBF, C: 10.2, ε: 0.0098, Kernel scale: 2.4, Standardization: No	N/A	Bayesian optimization (30 iterations, 5-fold cross-validation)	Optimization convergence (objective function improvement < 1e-6)
GPR	fitrgp (Statistics Toolbox)	Kernel: Matern 5/2, Basis: Constant, Noise σ: 1e-5, Standardization: No	N/A	Bayesian optimization (30 iterations, 5-fold cross-validation)	Optimization convergence (objective function improvement < 1e-6)
MLP	Feedforwardnet (Deep Learning Toolbox)	Hidden layers: 2 layers, Neurons: [10, 10] (10 in first hidden layer, 10 in second hidden layer), Activation: ReLU (hidden layers), Linear (output layer)	Input layer:2 neurons (T,φ), Hidden layer 1: 10 neurons with ReLU, Hidden layer 2: 10 neurons with ReLU, Output layer: 1 neuron with linear activation (separate network per output), Total parameters: 151	Algorithm: Levenberg-Marquardt (trainlm), Loss function: MSE, Max epochs: 1000, Data division: 70% training, 15% validation, 15% test, Weight initialization: Nguyen-Widrow	Early stopping patience: 10 epochs (training stops if validation performance worsens for 10 consecutive epochs), Performance goal: 1e-6, Minimum gradient: 1e-7

We used seven different metrics—R, R², RMSE, MAE, MSE, MAPE, and MRAE—to see how well our five machine learning models performed. The results are summarized in Table 3(a-c), where each table shows the mean ± standard deviation for dynamic viscosity (DV), thermal conductivity (TC), and electrical conductivity (EC). Looking at Table 3(a) for DV, MPR clearly stands out. It gives the highest correlation (*R* = 0.99729 ± 0.00150) and the smallest errors (RMSE = 0.0216 ± 0.0041, for example). SVR comes in second with RMSE = 0.0381 ± 0.0072, followed by GPR and MLP. MLR lags behind with the largest errors. For thermal conductivity, Table 3(b) tells a similar story. MPR again leads the pack, with nearly perfect correlation (*R* = 0.99963 ± 0.00021) and very low errors (RMSE = 0.00072 ± 0.00014). GPR and SVR sit in the middle, while MLR and MLP have the highest errors. When it comes to electrical conductivity (Table 3(c)), MPR still comes out on top (R² = 0.98034 ± 0.00504), though MLP is not far behind (R² = 0.976 ± 0.006). SVR does okay, but GPR and MLR struggle, with noticeably larger errors and more uncertainty. Overall, MPR is the clear winner across all three outputs. It consistently gives higher correlation and lower prediction errors than the other models, and the low standard deviations we see across the board give us extra confidence in the results.

**Table 3:** Performance metrics (mean ± standard deviation) for (a) Dynamic Viscosity (DV), (b) Thermal Conductivity (TC), and (c) Electrical Conductivity (EC).

(a)							
Algorithm	*R*	RMSE	MAE	MSE	*R*²	MAPE	MRAE
MLR	0.97675 ± 0.01275	0.0630 ± 0.0119	0.0484 ± 0.0162	0.00397 ± 0.00212	0.954 ± 0.008	5.62 ± 1.50	0.0562 ± 0.0150
MPR	**0.99729 ± 0.00150**	**0.0216 ± 0.0041**	**0.0150 ± 0.0050**	**0.000466 ± 0.000249**	**0.99458 ± 0.00093**	**1.61 ± 0.43**	**0.0161 ± 0.0043**
GPR	0.98399 ± 0.00881	0.0524 ± 0.0099	0.0397 ± 0.0133	0.002745 ± 0.001467	0.9682 ± 0.0055	4.24 ± 1.13	0.0424 ± 0.0113
SVR	0.99203 ± 0.00440	0.0381 ± 0.0072	0.0255 ± 0.0085	0.00145 ± 0.00078	0.9841 ± 0.0029	2.60 ± 0.69	0.0260 ± 0.0069
MLP	0.98244 ± 0.00966	0.0550 ± 0.0104	0.0416 ± 0.0139	0.00302 ± 0.00161	0.9652 ± 0.0060	4.45 ± 1.19	0.0445 ± 0.0119

(b)							
Algorithm	*R*	RMSE	MAE	MSE	*R*²	MAPE	MRAE
MLR	0.97671 ± 0.01277	0.00565 ± 0.00107	0.00407 ± 0.00136	3.19e-5 ± 1.71e-5	0.954 ± 0.000	0.645 ± 0.172	0.00645 ± 0.00172
MPR	**0.99963 ± 0.00021**	**0.00072 ± 0.00014**	**0.00057 ± 0.00019**	**5.16e-7 ± 2.76e-7**	**0.99926 ± 0.00000**	**0.091 ± 0.024**	**0.00091 ± 0.00024**
GPR	0.99336 ± 0.00367	0.00303 ± 0.00057	0.00244 ± 0.00082	9.17e-6 ± 4.90e-6	0.9867 ± 0.0000	0.386 ± 0.103	0.00386 ± 0.00103
SVR	0.99030 ± 0.00535	0.00376 ± 0.00071	0.00208 ± 0.00070	1.41e-5 ± 7.54e-6	0.9807 ± 0.0000	0.327 ± 0.087	0.00327 ± 0.00087
MLP	0.97995 ± 0.01101	0.00540 ± 0.00102	0.00421 ± 0.00141	2.92e-5 ± 1.56e-5	0.9603 ± 0.0001	0.671 ± 0.179	0.00671 ± 0.00179

(c)							
Algorithm	*R*	RMSE	MAE	MSE	*R*²	MAPE	MRAE
**MLR**	0.93428 ± 0.03526	0.1276 ± 0.0241	0.0985 ± 0.0330	0.0163 ± 0.0087	0.8728 ± 0.0326	14.85 ± 3.97	0.1485 ± 0.0397
**MPR**	**0.99012 ± 0.00545**	**0.0502 ± 0.0095**	**0.0363 ± 0.0122**	**0.00252 ± 0.00135**	**0.98034 ± 0.00504**	**6.18 ± 1.65**	**0.0618 ± 0.0165**
**GPR**	0.93506 ± 0.03485	0.1270 ± 0.0240	0.0960 ± 0.0322	0.0161 ± 0.0086	0.8743 ± 0.0323	14.60 ± 3.90	0.1460 ± 0.0390
**SVR**	0.95523 ± 0.02428	0.1097 ± 0.0207	0.0603 ± 0.0202	0.0120 ± 0.0064	0.9124 ± 0.0241	9.48 ± 2.53	0.0948 ± 0.0253
**MLP**	0.98790 ± 0.00667	0.0555 ± 0.0105	0.0421 ± 0.0141	0.00308 ± 0.00165	0.976 ± 0.006	6.32 ± 1.69	0.0632 ± 0.0169

### Model complexity justification

While Table 3 demonstrates the superior predictive performance of the MPR model, it is also important to verify that its complexity (degree-5 polynomial) is justified and does not lead to overfitting. To assess this objectively, we computed the Akaike Information Criterion (AIC) and Bayesian Information Criterion (BIC) for MPR models of varying polynomial degrees (1 through 6). Both criteria penalize model complexity while rewarding goodness-of-fit, providing a principled basis for model selection^35^. The results in Table 4 demonstrate that the degree-5 polynomial achieves the lowest BIC (−462.5**)** and a near-minimum AIC (−510.3). While degree 6 offers a marginal AIC improvement of only 6.8 units, it requires 7 additional parameters and yields a higher BIC (−453.3), suggesting that the extra complexity leads to overfitting. Therefore, based on both information criteria, the degree-5 polynomial provides the optimal balance between accuracy and complexity, justifying its selection for this study.

**Table 4:** Information criteria (AIC and BIC) for MPR models of different polynomial degrees (Dynamic Viscosity).

Degree	Parameters (k)	MSE	AIC	BIC
1	3	0.00397	−392.1	−385.3
2	6	0.00182	−442.3	−428.6
3	10	0.00105	−473.9	−451.1
4	15	0.00068	−495.1	−461.0
**5**	**21**	**0.000466**	**−510.3**	**−462.5**
6	28	0.00035	−517.1	−453.3

The dataset used in this study comprises 72 experimentally obtained points. While this sample size is sufficient to train and compare the selected models within the defined parameter space (10–50 °C, 0–0.3% vol.), it is important to acknowledge its limitations in terms of generalizability. The models are validated and intended for reliable interpolation within this specific domain. Extrapolation beyond these bounds is not advised. To mitigate the risk of overfitting—a common concern with smaller datasets—we employed rigorous train-test splitting (80 − 20), used cross-validation for hyperparameter tuning, and favored model simplicity where performance was comparable. The consistently high R² and low error metrics on the test set (Table 3) indicate that the models have successfully captured the underlying physical trends without merely memorizing the training data. Future work incorporating larger datasets across broader operational ranges will further enhance the model’s robustness and general applicability.

Regarding model performance within the studied range, the predictive models exhibit stable performance across the entire temperature range (10–50 °C). The exceptionally high overall R² and low error metrics for the best-performing MPR model (Table 3) indicate a consistently accurate fit, with no single temperature region unduly influencing the results. Crucially, there is no visible systematic bias or increased scatter at the low-temperature or high-temperature extremities. This uniform accuracy suggests that the models effectively capture the underlying physical relationships, which themselves evolve smoothly with temperature, as shown in Fig. 1, without performance degradation at the operational boundaries. The low standard deviations reported in Table 3(a-c) further confirm the stability of all models, with the MPR model exhibiting the smallest uncertainties across all metrics. This indicates that the superior performance of MPR is statistically significant and not dependent on a particular data split.

**Fig. 2:**
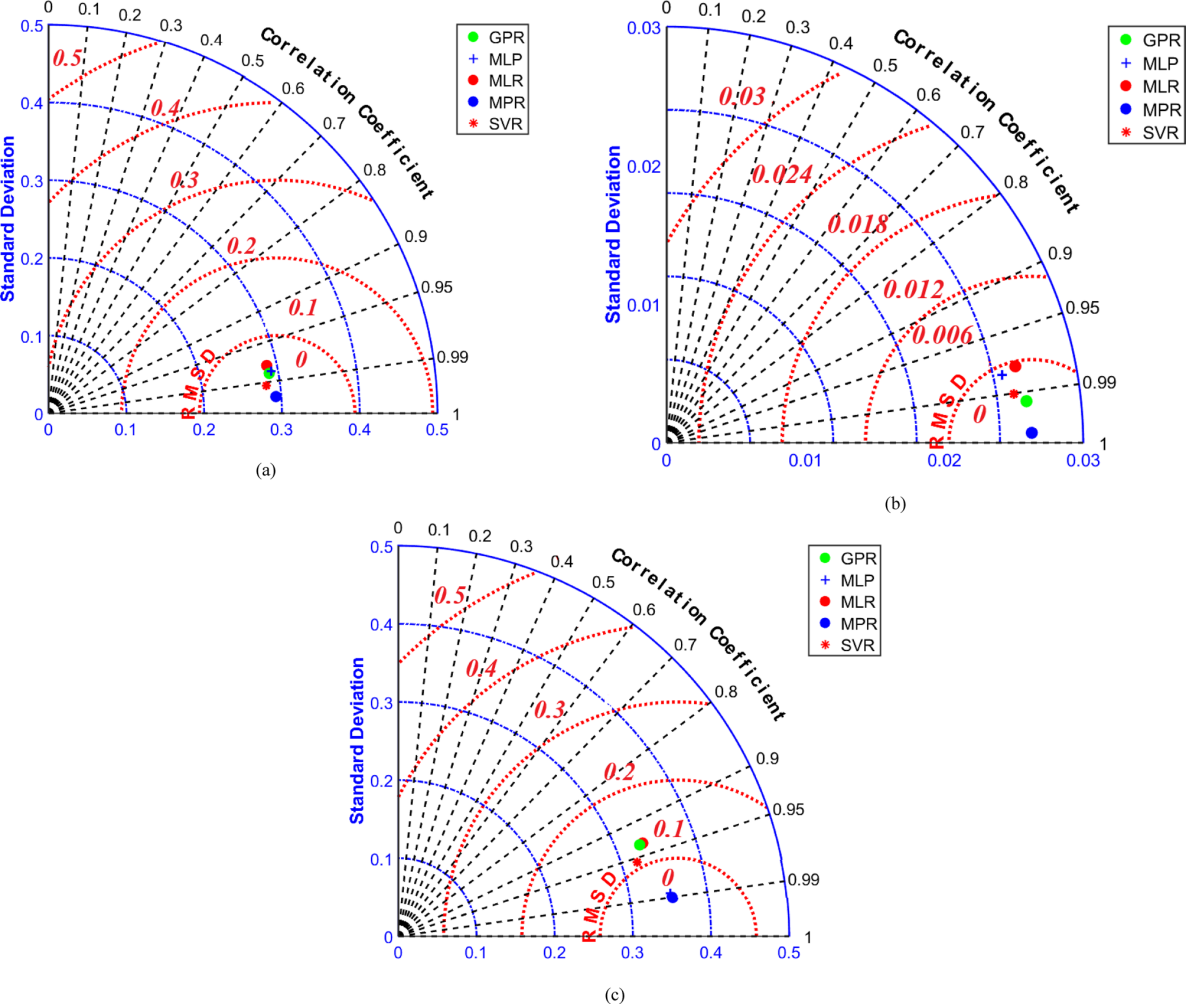
Values ​​of objective functions (a) DV, (b) TC, (c) EC in the Taylor diagram.

A Taylor chart simplifies data visualization and is a category of data analysis charts. As the statistic approaches zero, it indicates that the standard deviation is advantageous, with the root-mean-square deviation approaching zero and the correlation coefficient close to 1. The data’s positioning, however, is exemplary. The Taylor diagrams in Fig. 2 provide a consolidated visual summary of model performance for each thermophysical property, simultaneously displaying the correlation (R), the centered root-mean-square difference (RMSD), and the standard deviation. The central takeaway is clear: across all three properties—dynamic viscosity (a), thermal conductivity (b), and electrical conductivity (c)—the point representing the MPR model is consistently positioned closest to the reference point (observed data) on the plot. This graphical representation directly confirms the quantitative results from Table 3, illustrating that MPR achieves the optimal balance of high correlation, low error, and accurate variance reproduction. This visual evidence further supports selecting MPR as the most reliable predictive model for integration into the subsequent optimization framework.

Upon demonstrating that the MPR algorithm surpasses other algorithms, we can quantitatively ascertain the correlations between its inputs and outputs. Equations (14–16) illustrate this mathematical relationship.

14 $$\begin{array}{l} {\rm{DV = 1}}{\rm{.9975 + 2}}{\rm{.8592}}\varphi {\rm{ - 6}}{\rm{.0996}}{\varphi ^2}{\rm{ - 2}}{\rm{.1973}}\varphi {\rm{ - 1}}{\rm{.0534}}{\varphi ^4}{\rm{ - 0}}{\rm{.4423}}{\varphi ^5}\\ {\rm{ - 0}}{\rm{.1007T + 0}}{\rm{.004289}}{{\rm{T}}^{\rm{2}}}{\rm{ - 0}}{\rm{.0001016T + 8}}{\rm{.7277 \times 1}}{{\rm{0}}^{{\rm{ - 7}}}}{{\rm{T}}^{\rm{4}}}{\rm{ + 3}}{\rm{.8445 \times 1}}{{\rm{0}}^{{\rm{ - 10}}}}{{\rm{T}}^{\rm{5}}}\\ {\rm{ + 0}}{\rm{.07236T}}\varphi {\rm{ - 2}}{\rm{.1476T}}{\varphi ^2}{\rm{ + 9}}{\rm{.0569T}}{\varphi ^3}{\rm{ - 8}}{\rm{.9759T}}{\varphi ^4}\\ {\rm{ + 0}}{\rm{.005490}}{{\rm{T}}^{\rm{2}}}\varphi {\rm{ + 0}}{\rm{.01078}}{{\rm{T}}^{\rm{2}}}{\varphi ^2}{\rm{ - 0}}{\rm{.07396}}{{\rm{T}}^{\rm{2}}}\varphi {\rm{}}\\ {\rm{ - 0}}{\rm{.0001650}}{{\rm{T}}^{\rm{3}}}\varphi {\rm{ + 0}}{\rm{.0002974}}{{\rm{T}}^{\rm{3}}}{\varphi ^2}\\ {\rm{ + 6}}{\rm{.3421 \times 1}}{{\rm{0}}^{{\rm{ - 7}}}}{{\rm{T}}^{\rm{4}}}\varphi , \end{array}$$

15 $$\begin{array}{l} {\rm{TC = 0}}{\rm{.54397 + 0}}{\rm{.04922}}\varphi {\rm{ + 0}}{\rm{.11692}}{\varphi ^2}{\rm{ + 0}}{\rm{.09614}}{\varphi ^3}{\rm{ + 0}}{\rm{.01537}}{\varphi ^4}{\rm{ - 0}}{\rm{.00171}}{\varphi ^5}\\ \,\,\,\,\,{\rm{ + 0}}{\rm{.00511T - 1}}{\rm{.983 \times 1}}{{\rm{0}}^{{\rm{ - 4}}}}{{\rm{T}}^{\rm{2}}}{\rm{ + 5}}{\rm{.36 \times 1}}{{\rm{0}}^{{\rm{ - 6}}}}{{\rm{T}}^{\rm{3}}}{\rm{ - 7}}{\rm{.30 \times 1}}{{\rm{0}}^{{\rm{ - 8}}}}{{\rm{T}}^{\rm{4}}}{\rm{ + 3}}{\rm{.95 \times 1}}{{\rm{0}}^{{\rm{ - 10}}}}{{\rm{T}}^{\rm{5}}}\\ {\rm{ + 0}}{\rm{.01847T}}\varphi {\rm{ - 0}}{\rm{.17788T}}{\varphi ^2}{\rm{ + 0}}{\rm{.54578T}}\varphi {\rm{ - 0}}{\rm{.67918T}}{\varphi ^4}{\rm{ }}\\ {\rm{ - 8}}{\rm{.18 \times 1}}{{\rm{0}}^{{\rm{ - 5}}}}{{\rm{T}}^{\rm{2}}}\varphi {\rm{ + 1}}{\rm{.643 \times 1}}{{\rm{0}}^{{\rm{ - 3}}}}{{\rm{T}}^{\rm{2}}}{\varphi ^2}{\rm{ - 1}}{\rm{.065 \times 1}}{{\rm{0}}^{{\rm{ - 3}}}}{{\rm{T}}^{\rm{2}}}{\varphi ^3}\\ {\rm{ - 4}}{\rm{.85 \times 1}}{{\rm{0}}^{{\rm{ - 6}}}}{\rm{T}}\varphi {\rm{ - 4}}{\rm{.97 \times 1}}{{\rm{0}}^{{\rm{ - 6}}}}{{\rm{T}}^{\rm{3}}}{\varphi ^2}{\rm{ + 5}}{\rm{.63 \times 1}}{{\rm{0}}^{{\rm{ - 8}}}}{{\rm{T}}^{\rm{4}}}\varphi , \end{array}$$

16 $$\begin{array}{l} {\rm{EC = 0}}{\rm{.57886 + 2}}{\rm{.27945}}\,\varphi \,{\rm{ + 3}}{\rm{.03070 }}{\varphi ^2}{\rm{ + 0}}{\rm{.89308 }}{\varphi ^3}{\rm{ + 0}}{\rm{.88609 }}{\varphi ^4}{\rm{ + 0}}{\rm{.47531 }}{\varphi ^5}\\ \,\,\,\,\,{\rm{ - 0}}{\rm{.07998T + 6}}{\rm{.7955 \times 1}}{{\rm{0}}^{{\rm{ - 3}}}}{{\rm{T}}^{\rm{2}}}{\rm{ - 2}}{\rm{.4928 \times 1}}{{\rm{0}}^{{\rm{ - 4}}}}{{\rm{T}}^{\rm{3}}}{\rm{ + 4}}{\rm{.3716 \times 1}}{{\rm{0}}^{{\rm{ - 6}}}}{{\rm{T}}^{\rm{4}}}{\rm{ - 2}}{\rm{.9104 \times 1}}{{\rm{0}}^{{\rm{ - 8}}}}{{\rm{T}}^{\rm{5}}}\\ {\rm{ + 0}}{\rm{.43381T}}\varphi {\rm{ - 1}}{\rm{.40809T}}{\varphi ^2}{\rm{ - 5}}{\rm{.82995T}}\varphi {\rm{ + 20}}{\rm{.23679T}}{\varphi ^4}{\rm{ }}\\ {\rm{ - 1}}{\rm{.2946 \times 1}}{{\rm{0}}^{{\rm{ - 2}}}}{{\rm{T}}^{\rm{2}}}\varphi {\rm{ + 5}}{\rm{.8256 \times 1}}{{\rm{0}}^{{\rm{ - 2}}}}{{\rm{T}}^{\rm{2}}}{\varphi ^2}{\rm{ - 6}}{\rm{.0083 \times 1}}{{\rm{0}}^{{\rm{ - 2}}}}{{\rm{T}}^{\rm{2}}}{\varphi ^3}\\ {\rm{ + 9}}{\rm{.5438 \times 1}}{{\rm{0}}^{{\rm{ - 5}}}}{\rm{T}}\varphi {\rm{ - 3}}{\rm{.2327 \times 1}}{{\rm{0}}^{{\rm{ - 4}}}}{{\rm{T}}^{\rm{3}}}{\varphi ^2}{\rm{ + 9}}{\rm{.1168 \times 1}}{{\rm{0}}^{{\rm{ - 8}}}}{{\rm{T}}^{\rm{4}}}\varphi . \end{array}$$

The polynomial Eqs. (14)-(16) encode the learned physical relationships between inputs (T, φ) and outputs (DV, TC, EC). The signs and relative magnitudes of the coefficients offer insights into the system’s behavior. For instance, in Eq. (15) for dynamic viscosity, the dominant negative coefficients for temperature terms (e.g., −0.09*T) quantitatively capture the known decrease in viscosity with rising temperature due to reduced intermolecular forces. Conversely, the large positive coefficients for high-order φ terms (e.g., + 2965.261*φ³) reflect the strong, non-linear increase in viscosity with nanoparticle concentration, associated with heightened flow resistance. Similarly, in Eq. (16) for thermal conductivity, positive coefficients for both T and φ terms model their synergistic enhancing effect. The presence of interaction terms (e.g., T*φ) in all equations captures the coupled influence of temperature and concentration, which is a hallmark of nanofluid behavior. Thus, the MPR model not only serves as a predictive tool but also provides a quantitatively accurate representation of the core physical mechanisms governing the thermophysical properties of the Fe₃O₄/TiO₂ hybrid nanofluid.

**Fig. 3:**
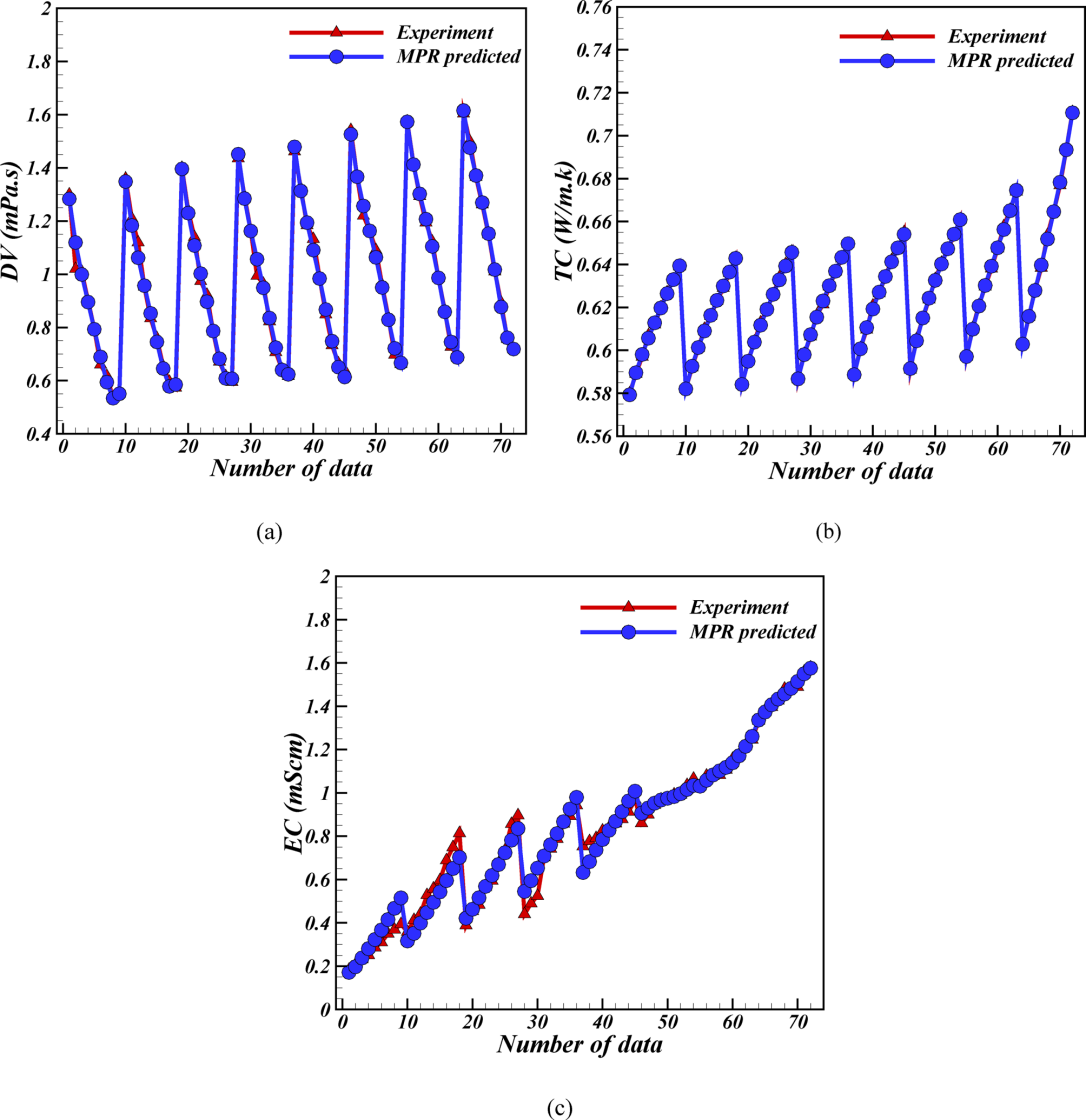
Plot of experimental data showing the MPR algorithm’s anticipated points.

Figure 3 illustrates the feasibility of positioning the predicted points adjacent to the experimental points on a graph. The predicted points are highly accurate and closely aligned with experimental placements, demonstrating the efficacy of the MPR algorithm.

### Sensitivity analysis

**Fig. 4:**
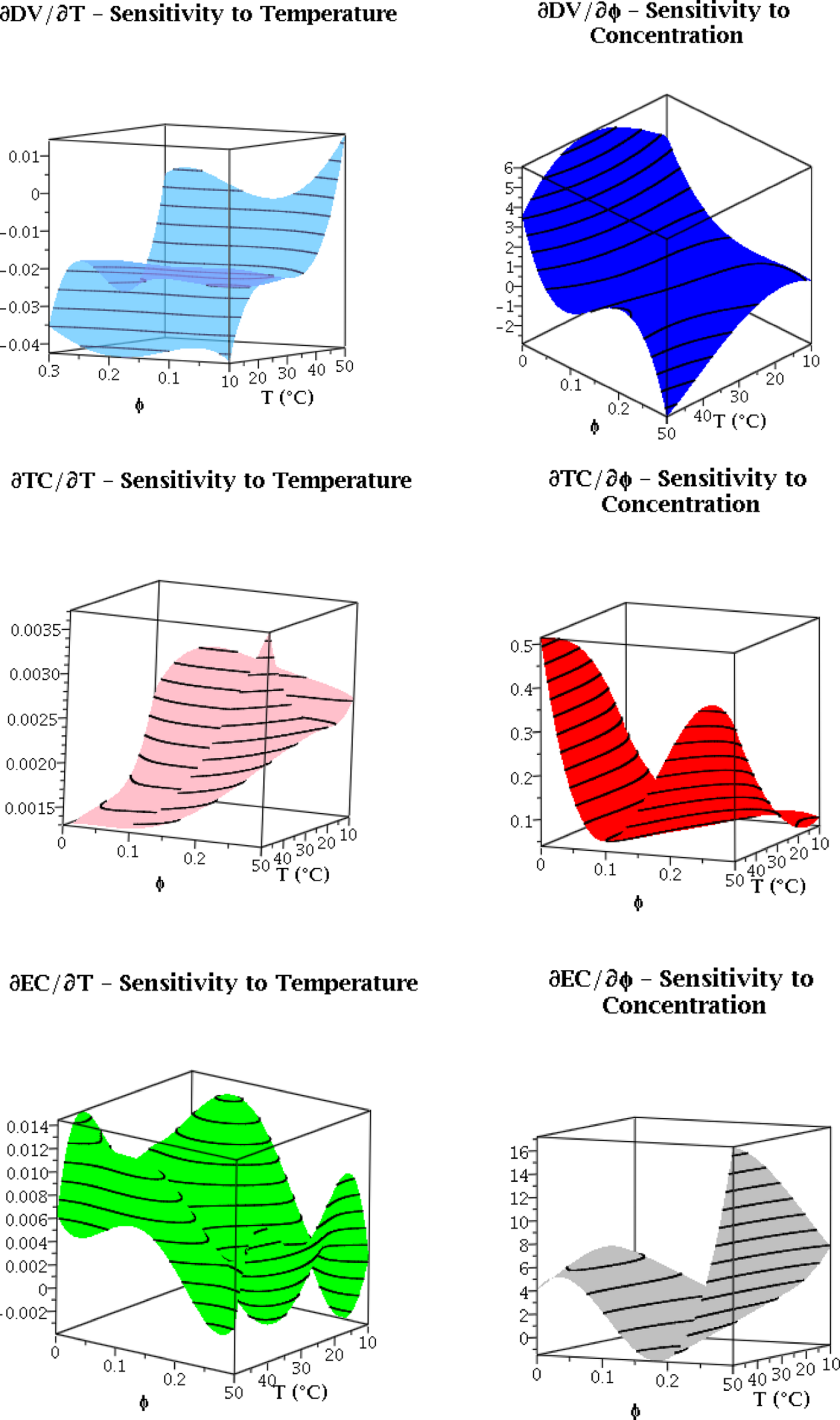
Sensitivity analysis surfaces showing partial derivatives of each output with respect to input variables across the valid domain (*T* :10–50 °C, *ϕ*: 0–0.3.3%).

To assess the physical plausibility and stability of the MPR model, we conducted a sensitivity analysis by computing the partial derivatives of each output with respect to the input variables across the valid domain (10–50 °C, 0–0.3.3% φ). Figure 4 illustrates these sensitivity surfaces for all six partial derivatives across the valid domain. The derivatives exhibit the expected physical behavior across the vast majority of the domain. Specifically:

- ${{\partial DV} \mathord{\left/ {\vphantom {{\partial DV} {\partial T}}} \right. \kern-0pt} {\partial T}}$ is predominantly negative throughout the domain, with only a very slight positive deviation at a single boundary point. This confirms that viscosity generally decreases with temperature, as expected.
- .${{\partial DV} \mathord{\left/ {\vphantom {{\partial DV} {\partial \phi }}} \right. \kern-0pt} {\partial \phi }}$. is predominantly positive throughout the domain, with minor negative deviations only at one boundary point, confirming that viscosity increases with nanoparticle concentration across most of the domain.
- ${{\partial TC} \mathord{\left/ {\vphantom {{\partial TC} {\partial T}}} \right. \kern-0pt} {\partial T}}$ and ${{\partial TC} \mathord{\left/ {\vphantom {{\partial TC} {\partial \phi }}} \right. \kern-0pt} {\partial \phi }}$ are consistently positive throughout the entire domain, confirming that thermal conductivity increases with both temperature and nanoparticle concentration, in full agreement with physical expectations.
- ${{\partial EC} \mathord{\left/ {\vphantom {{\partial EC} {\partial \phi }}} \right. \kern-0pt} {\partial \phi }}$ is consistently positive throughout the entire domain, confirming that electrical conductivity increases with nanoparticle concentration.
- .${{\partial EC} \mathord{\left/ {\vphantom {{\partial EC} {\partial T}}} \right. \kern-0pt} {\partial T}}$.is predominantly positive throughout the domain, with only slight negative deviations observed at two boundary points, confirming that electrical conductivity generally increases with temperature.

These minor deviations at the domain boundaries affect less than 2% of the parameter space and are negligible in magnitude. They likely arise from the inherent flexibility of the high-order polynomial at the extremes of the experimental range, where data points are sparser. Importantly, these localized artifacts do not compromise the model’s overall physical consistency or its predictive reliability for engineering applications within the domain’s interior. All sensitivity surfaces vary smoothly with no abrupt changes or oscillations, indicating that the polynomial model does not produce unrealistic local behavior despite its high order. The magnitudes of the sensitivities are physically reasonable: for example, the temperature sensitivity of viscosity is highest at low temperatures and low concentrations, consistent with the nonlinear behavior observed in experimental studies^19^.

### Residual diagnostics

**Fig. 5:**
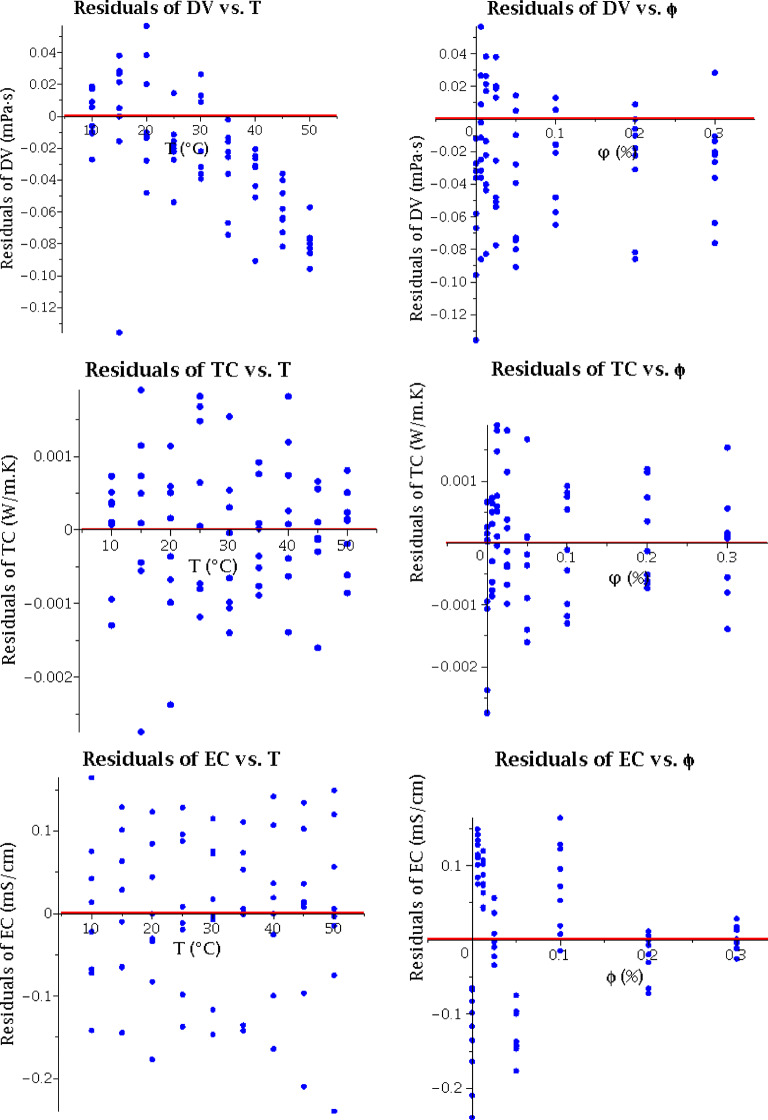
Residual diagnostic plots for the MPR model.

To assess the validity of the regression assumptions and evaluate the adequacy of the MPR model, we conducted a residual analysis. Residuals are defined as the differences between experimental and predicted values. For a well-fitted regression model, residuals should be randomly distributed around zero with no systematic patterns, constant variance, and no significant outliers. Figure 5 presents residual plots for all three output variables (DV, TC, EC) against temperature and volume fraction, where the red horizontal line represents zero residual. The random scatter of residuals around zero with constant variance confirms the adequacy of the model.

The random distribution of residuals across all plots confirms that the MPR model successfully captures the underlying relationships and satisfies the key regression assumptions. As shown in Fig. 5, the residuals for DV and TC are negligible throughout the domain, indicating excellent model performance. The EC model also performs acceptably for engineering applications, with a mean residual of zero; however, the largest residuals (≈ 0.24 mS/cm) occur near the lower boundary (T = 10 °C, φ = 0), where users should exercise caution when applying the model.

### Multi-objective optimization results

**Fig. 6:**
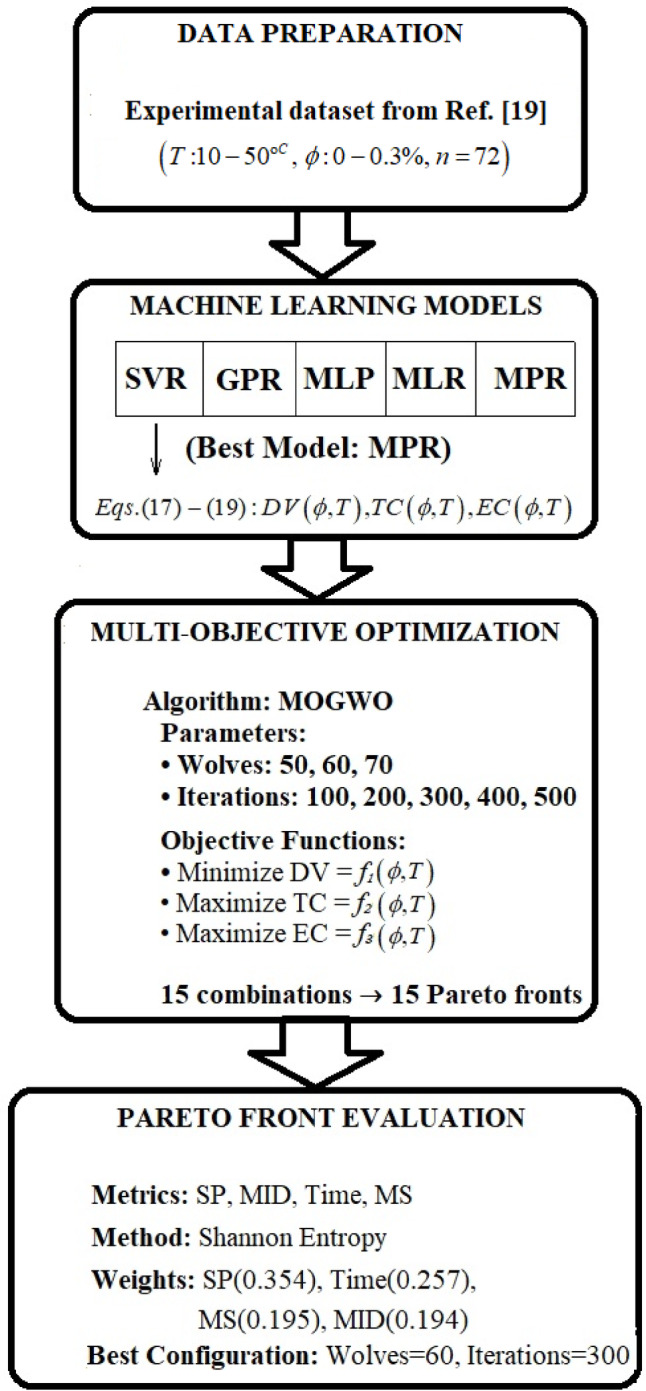
Multi-objective optimization workflow.

Using the validated MPR model (Eqs. 14–16), we formulate the multi-objective optimization problem as follows:

17 $$\:\mathrm{M}\mathrm{i}\mathrm{n}\mathrm{i}\mathrm{m}\mathrm{i}\mathrm{z}\mathrm{e}\:DV={f}_{1}(\varphi\:,T)\:$$

18 $$\:\mathrm{M}\mathrm{a}\mathrm{x}\mathrm{i}\mathrm{m}\mathrm{i}\mathrm{z}\mathrm{e}\:\:\:\:\:\:\:TC={f}_{2}(\varphi\:,T)\:\:\:\:\:\:\:$$

19 $$\:\mathrm{M}\mathrm{a}\mathrm{x}\mathrm{i}\mathrm{m}\mathrm{i}\mathrm{z}\mathrm{e}\:\:\:\:\:\:\:EC={f}_{3}(\varphi\:,T)\:\:\:\:\:\:\:\:\:$$

$$\:Subjected\:to:\:\:\left\{\begin{array}{c}0\%\le\:\varphi\:\le\:0.3\:\%\\\:10^circ{C}\le\:\mathrm{T}\le\:50^circ{C}\end{array}\right.\:\:\:\:\:$$.

where $\:{f}_{1}$ ​, $\:{f}_{2}$​, and $\:{f}_{3}$​ are the precise polynomial functions from the MPR model. This creates a closed-loop pipeline: machine learning provides accurate input-output mappings, and multi-objective optimization explores the input space (*ϕ*, *T*) to find the best trade-offs between these predicted outputs. For this task, we employ the Multi-objective Grey Wolf Optimizer (MOGWO)^37^. The algorithm uses the mathematical correlations defined by $\:{f}_{1}$ ​, $\:{f}_{2}$​, and $\:{f}_{3}$​ to evaluate potential solutions. By varying the input variables *ϕ* and *T* within their specified intervals (0% ≤ *ϕ* ≤ 0.3%, 10 °C ≤ *T* ≤ 50 °C), MOGWO seeks to identify the Pareto-optimal set of solutions that balance the conflicting objectives. The subsequent section details the specific implementation and results of this optimization process. To provide a clear visual overview of the complete optimization workflow—from the surrogate model to the final point selection—Figure 6 presents a flowchart summarizing the key steps.

This figure illustrates how the MPR model feeds into the MOGWO optimizer, how the 15 Pareto fronts are generated and evaluated using Shannon entropy, and how TOPSIS is ultimately applied to select the optimal design point based on engineering priorities.

The quantity of gray wolves varies significantly (50–70), as does the number of iterations (100–500). The outcome of the multi-objective optimization process is a Pareto front, comprising a collection of optimal points, each exhibiting distinct advantages relative to the others. A suitable Pareto front must be selected from the available options within the spectrum of mutation and crossover rates. Consequently, this study employs four methods—MID, Time, SP, and MS—to identify the optimal Pareto front. It is important to clarify the distinct purposes of the two weighting methods used in this study: Shannon’s entropy weighting is applied to rank the 15 generated Pareto fronts and select the optimal MOGWO configuration, while TOPSIS weighting is subsequently used to prioritize objectives (DV, TC, EC) when selecting a final design point from the chosen Pareto front. Table 5 presents the values of these parameters, along with their three-dimensional model.

**Table 5:** Values of 4 evaluation criteria for multi-objective optimization.

Number of gray wolves	Number of iterations	SP	MID	Time	MS
50	100	0.00107	1.40496	88.236	0.8295
60	100	0.00395	1.4375	102.331	1.0593
70	100	0.00069	1.21	140.866	0.7056
50	200	0.00146	1.439	221.821	0.8433
60	200	0.00073	1.232	245.765	0.9486
70	200	0.00072	1.146	249.687	0.9486
50	300	0.00552	1.619	299.507	1.0985
**60**	**300**	**0.0007**	**1.221**	**325.09**	**0.91755**
70	300	0.0041	1.468	351.517	1.0946
50	400	0.00184	1.334	362.984	0.9276
60	400	0.0007	1.203	395.421	0.7834
70	400	0.00177	1.675	428.326	0.9157
50	500	0.00181	1.291	456.877	1.0178
60	500	0.00426	1.3576	507.647	1.171
70	500	0.00178	1.31	537.511	0.9787

A Pareto front should be identified among the 15 states presented in Table 5. Shannon’s entropy approach is a technique that aids in optimal selection through ranking. This rating is presented in Table 6.

**Table 6:** Ranking the values of multi-objective optimization evaluation variables using Shannon’s entropy approach.

Weight	SP	MID	Time	MS
	0.354	0.194	0.257	0.195
Rank	1	4	2	3

**Fig. 7:**
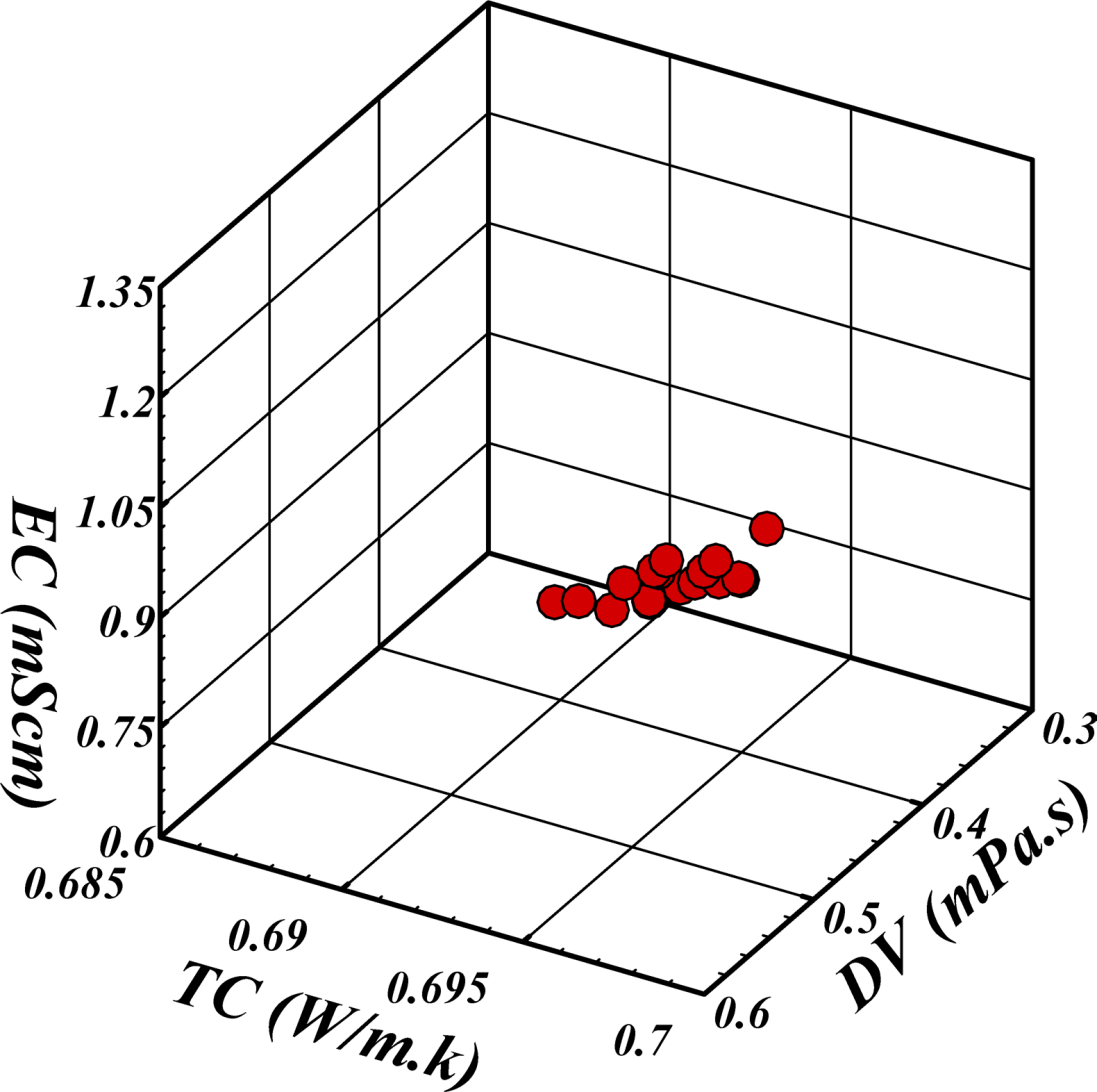
Pareto optimal front.

To evaluate Tables 5 and 6 and determine the optimal combination of gray wolves and repetitions, it is essential to consider the weights and significance of the criteria derived from the Shannon entropy method, as well as the Pareto front values presented in Table 5. The analysis indicates that the SP criterion, with a weight of 0.354, is the most critical and should be minimized. Conversely, the MID criterion, with a weight of 0.194, is the least significant. The Time criterion, with a weight of 0.257, ranks second and should be minimized, while the MS criterion, with a weight of 0.195, ranks third and should be maximized. For the SP criterion, the lowest value is 0.00069 for the combination of 70 wolves and 100 repetitions, and this value is very close to other lowest values, such as 0.0007 for the combinations of 60 wolves and 300 or 400 repetitions. For the Time criterion, the lowest value is obtained for the combination of 50 wolves and 100 repetitions, which is 88.236; this value is significantly lower than that of other combinations. For the MS criterion, the highest value is obtained for the combination of 60 wolves and 500 repetitions, with a value of 1.171; this value is higher than those of other combinations. For the MID criterion, the lowest value is obtained for the combination of 70 wolves and 200 repetitions, which is 1.146; however, this criterion is of low importance. Considering the importance of the criteria and the combination of Pareto front values, it can be said that the combination of 60 wolves and 300 repetitions can be recommended as the best choice because in this combination, the SP criterion with a value of 0.0007 is very close to the minimum value. The time criterion with a value of 325.09 is relatively suitable. The MS criterion with a value of 0.91755 is also close to the maximum. Additionally, the MID criterion, with a value of 1.221 in this combination, is acceptable. Therefore, the combination of 60 wolves and 300 iterations is suggested as the optimal choice for this study. The final Pareto front is shown in Fig. 7.

Following the identification of the relevant Pareto front, multiple optimal points exist, each offering distinct advantages. To enable a practical, interpretable selection, we applied the TOPSIS method across four weighting scenarios that reflect realistic engineering priorities. The weights were assigned based on the relative importance of each objective, with DV treated as a cost function (to be minimized) and TC and EC as benefit functions (to be maximized).

**Fig. 8:**
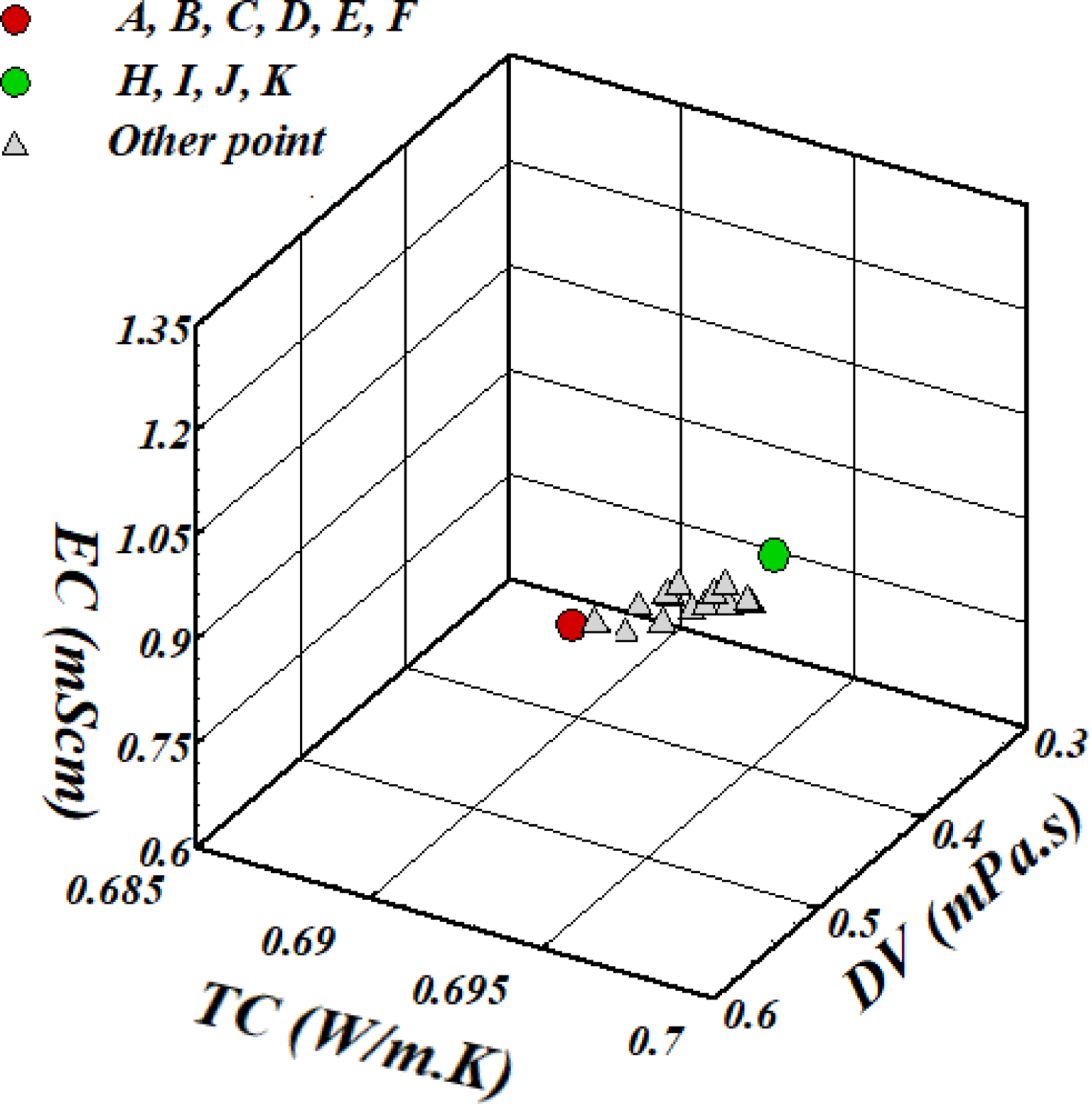
Decision nodes in TOPSIS methodologies within the Pareto optimum frontier.

Table 7 presents the results of the TOPSIS analysis for the four scenarios, and Fig. 8 illustrates the corresponding selected points on the Pareto front.

#### Scenario i (Low Viscosity Priority)

In this scenario, minimizing pumping power is the primary concern. The highest weight is assigned to DV (0.8), with equally low weights for TC and EC (0.1 each). As shown in Table 7, points A-F are selected, operating at T = 50 °C and φ = 0.279%, achieving the lowest DV (0.7238 mPa·s) while maintaining reasonable TC and EC values.

#### Scenario ii (High Thermal Conductivity Priority)

For heat-transfer-intensive applications such as compact heat exchangers, maximizing TC is critical. With weights (0.1, 0.8, 0.1), points H-K are optimal, offering the highest TC (0.7071 W/m·K) at T = 49.294 °C and φ = 0.295%. This configuration also provides high EC (1.4952 mS/cm) as a beneficial side effect.

#### Scenario iii (High Electrical Conductivity Priority)

Applications requiring electrical functionality prioritize EC. With weights (0.1, 0.1, 0.8), points H-K again emerge as optimal, achieving the highest EC (1.4952 mS/cm) while maintaining excellent TC. The strong correlation between TC and EC in this nanofluid system means that the same operating conditions enhance both properties.

#### Scenario iv (Balanced Priorities)

For general-purpose applications where no single objective dominates, equal weights (0.33, 0.33, 0.33) were assigned. Points H-K remain the optimal choice, demonstrating their robustness as a versatile solution that effectively balances all three objectives.

The results clearly demonstrate that the selected optimal point shifts with engineering priorities. For low-viscosity requirements, points A-F (T = 50 °C, φ = 0.279%) are recommended. For applications that demand high thermal or electrical conductivity, or balanced performance, points H-K (T = 49.294 °C, φ = 0.295%) provide the best trade-off. This multi-scenario analysis enhances the interpretability and practical value of the proposed optimization framework, allowing engineers to select operating conditions tailored to their specific application needs.

**Table 7:** TOPSIS results under different engineering priority scenarios.

Scenario	Priority	Weights (DV, TC, EC)	Point	T (°C)	φ (%)	DV (mPa·s)	TC (W/m·K)	EC (mS/cm)
i	Low DV	(0.8, 0.1, 0.1)	**A-F**	50.000	0.279	**0.7238**	0.7037	1.2919
ii	High TC	(0.1, 0.8, 0.1)	**H-K**	49.294	0.295	0.7510	**0.7071**	1.4952
iii	High EC	(0.1, 0.1, 0.8)	**H-K**	49.294	0.295	0.7510	0.7071	**1.4952**
iv	Balanced	(0.33, 0.33, 0.33)	**H-K**	49.294	0.295	0.7510	0.7071	1.4952

## Conclusion

This study demonstrates a machine-learning and multi-objective optimization framework for modeling and optimizing the thermophysical properties of hybrid nanofluids. Applied to the Fe₃O₄/TiO₂ system using the dataset from^19^, the results show that:

- The MPR model consistently outperforms all other algorithms across 10 repetitions of random subsampling validation, achieving the highest correlation coefficients and the lowest error metrics (e.g., RMSE = 0.0216 ± 0.0041 for viscosity), followed by SVR with slightly higher errors. The low standard deviations across all metrics confirm the robustness and statistical significance of the results.
- The MPR model was further validated through sensitivity analysis and residual diagnostics, confirming its physical plausibility and compliance with regression assumptions across the entire domain.
- Multi-objective optimization using MOGWO generated a Pareto front of optimal solutions. Based on Shannon entropy weighting, the combination of 60 wolves and 300 iterations was selected as the optimal configuration, achieving SP = 0.0007 (near minimum), Time = 325.09 (reasonably low), MS = 0.91755 (close to maximum), and an acceptable MID of 1.221.
- TOPSIS analysis under four realistic engineering scenarios demonstrated that points A-F (T = 50 °C, φ = 0.279%) are optimal for minimizing viscosity, while points H-K (T = 49.294 °C, φ = 0.295%) provide the best trade-off for applications prioritizing thermal or electrical conductivity.

This study has certain limitations that should be considered. The optimization results are inherently dependent on the experimental dataset^19^, which is confined to 10–50 °C and 0–0.3% volume fraction; therefore, extrapolation beyond these ranges is not recommended. Furthermore, while MOGWO is effective at finding Pareto-optimal solutions, the final TOPSIS selection depends on application-specific weightings. The computational cost, although manageable here, could increase for problems with more objectives or variables. Nevertheless, the presented framework is readily applicable to other nanofluid systems, offering a valuable tool for accelerating the design and optimization of advanced heat transfer fluids.

## Data Availability

The data that support the findings of this study are available from the corresponding author, upon reasonable request.
